# P-1605. Matching the Right Test to the Right Patient: Building Probability-Based Decision Support into the Electronic Health Record to Guide Diagnosis of Community-Acquired Pneumonia (CAP) Among Hospitalized Patients

**DOI:** 10.1093/ofid/ofae631.1772

**Published:** 2025-01-29

**Authors:** Jonathan Baghdadi, Anthony Harris, Danica Palacio, Drew Charles, Emily L Heil, Kimberly C Claeys, Jacqueline T Bork, Sarah Sommerkamp, R Gentry Wilkerson, Gerald Godwin, Mark Sutherland, Melinda M Neuhauser, Sarah Kabbani, Daniel J Morgan

**Affiliations:** University of Maryland School of Medicine, Baltimore, Maryland; University of Maryland School of Medicine, Baltimore, Maryland; University of Maryland School of Medicine, Baltimore, Maryland; Medical University of South Carolina , Charleston, South Carolina; University of Maryland School of Pharmacy, Baltimore, MD; University of Maryland Baltimore, Baltimore, Maryland; University of Maryland School of Medicine, Baltimore, Maryland; University of Maryland School of Medicine, Baltimore, Maryland; University of Maryland School of Medicine, Baltimore, Maryland; University of Maryland Medical System, Baltimore, Maryland; University of Maryland School of Medicine, Baltimore, Maryland; Division of Healthcare Quality Promotion, Centers for Disease Control and Prevention,, Atlanta, GA; Centers for Disease Control and Prevention, Atlanta, Georgia; University of Maryland School of Medicine, Baltimore, Maryland

## Abstract

**Background:**

Respiratory infections are among the most common reasons antibiotics are prescribed. However, 30-50% of patients started on antibiotics for CAP do not have bacterial infections.

**Figure:**
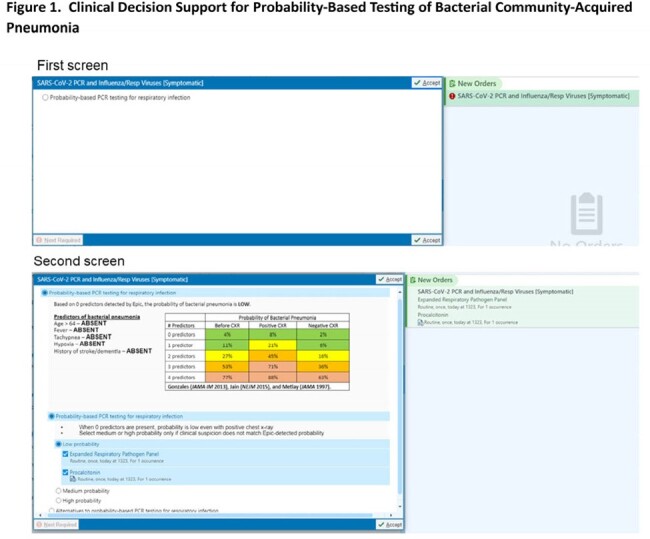

**Methods:**

We implemented clinical decision support (CDS) in the electronic health record of two hospitals to support diagnosis of viral vs. bacterial CAP. The CDS (1) estimates the probability of bacterial CAP using logic from a publicly available calculator and (2) provides probability-based options for diagnostic testing. The CDS is triggered by an order for respiratory virus testing and can be dismissed if bacterial CAP is not suspected or the patient is not being admitted. Design of the CDS was refined iteratively in usability testing with frontline clinicians (Fig. 1). The final design was an order panel embedded within other order sets (Fig. 2). The CDS was activated on 11/1/2023.

Impact of the CDS was assessed in a quasi-experiment comparing antibiotic use (AU) during the post-intervention respiratory virus season (10/2023 – 3/2024) to corresponding months in 2021-2022 and 2022-2023. AU was defined as days of therapy / 1000 patient bed-days (DOT/1000 BD) for antibiotics started on hospital day 0-7 for respiratory infection, regardless of whether the CDS triggered. AU categories, including extended-spectrum AU, were defined by pharmacy. Average quarterly AU was compared between the post-intervention respiratory virus season and preceding seasons using Student’s t-test.

**Figure:**
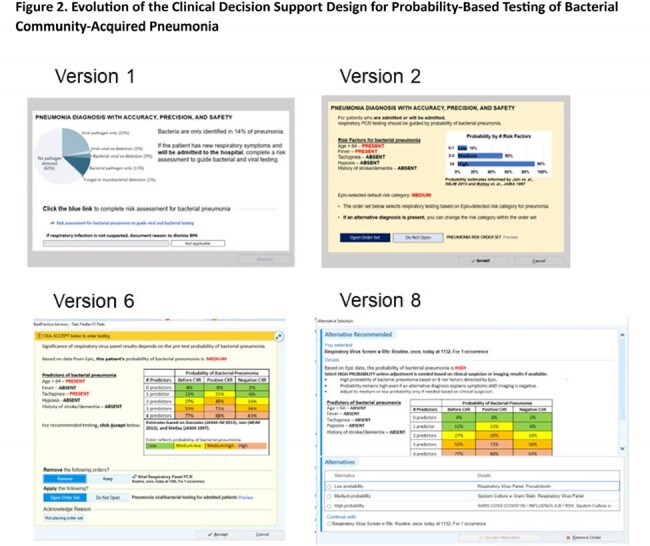

**Results:**

CDS guided test ordering in 890 of 3,201 eligible CAP patients (28% of appearances, Fig. 3) through 3/31/2024, with usage declining in February and March. Feedback from clinicians noted that the CDS is unobtrusive, but it does not apply to all patients, and recommended tests sometimes have longer turnaround times than alternatives. Overall AU for respiratory infections did not change in the post-intervention respiratory season (129.1 vs. 129.6 DOT/1000 BD, p = 0.94), but extended-spectrum AU decreased significantly (46.6 vs. 40.6 DOT/1kBD, p = 0.046, Fig. 4).

**Figure:**
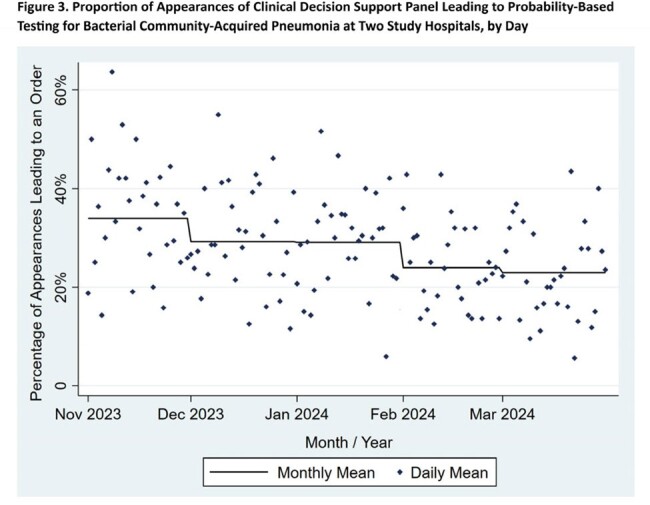

**Conclusion:**

This project demonstrates use of CDS to guide diagnostic testing based on pre-test probability of bacterial CAP. An associated antimicrobial stewardship intervention is under investigation (NCT05976581).

**Figure:**
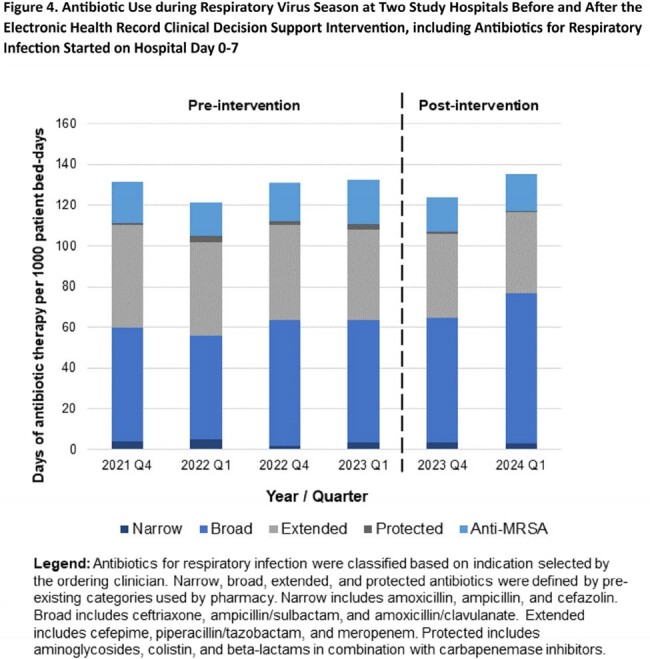

**Disclosures:**

**Anthony Harris, MD, MPH**, Innoviva: Advisor/Consultant|UpToDate: Infection Control Editor **Kimberly C. Claeys, PharmD, PhD**, bioMérieux: Advisor/Consultant|bioMérieux: Honoraria **R. Gentry Wilkerson, MD**, Beckton Dickinson: Grant/Research Support|CalciMedica: Grant/Research Support|CoapTech: Grant/Research Support|Eldon: Grant/Research Support|EndPoint Health, Inc: Grant/Research Support|Global Blood Therapeutics: Grant/Research Support|Novartis: Grant/Research Support|Pfizer: Grant/Research Support|Tonix: Grant/Research Support|Vapotherm: Grant/Research Support

